# Gut microbiota and traumatic brain injury: insights from an antibiotic-free cohort

**DOI:** 10.3389/fmicb.2025.1697206

**Published:** 2026-01-02

**Authors:** Hao Wu, Yuqi Huo, Wei Fang, Jinlin Guo, Xiaoqin Wang, Li Su, Gang Cheng

**Affiliations:** 1 Department of Neurosurgery, Shanxi Bethune Hospital, Shanxi Academy of Medical Sciences, Third Hospital of Shanxi Medical University, Tongji Shanxi Hospital, Taiyuan, Shanxi, China; 2 Department of Surgical Intensive Care Unit, Heping Campus of Shanxi Provincial People's Hospital, Taiyuan, Shanxi, China; 3 Department of Clinical Laboratory, Heping Campus of Shanxi Provincial People's Hospital, Taiyuan, Shanxi, China; 4 Department of Pharmacy, Shanxi Provincial People's Hospital, Taiyuan, Shanxi, China; 5 Department of Neurosurgery, Shenzhen University Affiliated South China Hospital, Shenzhen, Guangdong, China; 6 Department of Neurosurgery, Shanxi Provincial People's Hospital, Taiyuan, Shanxi, China

**Keywords:** traumatic brain injury, gut microbiota, short-chain fatty acid, functional prediction, biofilm, machine learning modeling

## Abstract

**Introduction:**

Traumatic brain injury (TBI) is a major global health concern, leading to persistent neurological deficits and systemic complications. While animal studies have shown the association between TBI and the gut microbiota, human evidence, particularly in the early post-injury period, remains scarce.

**Methods:**

In this study, we profiled the gut microbiota of TBI patients within 8 days of hospitalization using 16S rRNA sequencing, integrating clinical metadata and excluding individuals who had received antibiotics within the preceding month.

**Results:**

Although alpha diversity remained similar between groups, beta diversity was significantly altered in TBI patients relative to healthy controls, accompanied by increased inter-individual variability. Differential abundance analysis revealed a depletion of short-chain fatty acid (SCFA)-producing taxa, e.g., *Eubacterium, Agathobacter*, and *Faecalibacillus intestinalis*, alongside an enrichment of dysbiosis-associated genera, including *Escherichia* and *Shigella*. Notably, *Lactobacillus*, a γ-aminobutyric acid-producing genus, was elevated in TBI patients, potentially reflecting a compensatory response to neural injury. Functional prediction suggested reduced SCFA biosynthetic capacity in TBI patients, whereas biofilm formation and several other fitness-related processes were enriched. Integrating gut microbiota with clinical and demographic variables, a machine learning model moderately predicted Glasgow Coma Scale scores, with age, inflammatory markers, and differentially abundant bacterial taxa as major contributors.

**Discussion:**

Collectively, these findings indicate that TBI is associated with dysbiosis of the gut microbial and altered metabolic potential.

## Introduction

In 2021, the global incidence of traumatic brain injury (TBI) was estimated at approximately 259 new cases per 100,000 population, translating to over 20 million new TBI cases worldwide (Perin et al., 2022). Individuals with a history of TBI frequently experience significant impairments in lower extremity mobility and functional activities (Schneider et al., 2021). Moreover, survivors of TBI face heightened risks of induced diseases, e.g., post-traumatic epilepsy, neurodegenerative conditions like Alzheimer's and Parkinson's disease, various psychiatric disorders, hormonal imbalances from pituitary dysfunction, and chronic sleep disorders (Schneider et al., 2021).

There are several metabolites in the gut microbiome that have been shown to influence TBI. Short-chain fatty acids (SCFAs), notably butyrate, acetate, and propionate, are key microbial metabolites that influence immune and neurological outcomes (Guo et al., 2022; Lan et al., 2024). In a TBI mouse model, SCFA supplementation restores microglial homeostasis, supports T-cell trafficking into the brain, and promotes neurogenesis, implying a protective role of SCFA-producers in the gut microbiota in TBI (Celorrio et al., 2025). Meanwhile, γ-aminobutyric acid (GABA), synthesized by gut bacteria, e.g., *Bifidobacterium adolescentis*, acts as a neuroactive postbiotic that modulates neural signaling (Mazzoli and Pessione, 2016; Duranti et al., 2020). Although direct data linking GABA to TBI outcomes remain limited, its broader role in gut-brain communication, including regulation of anxiety, stress, and central neurotransmission, suggests potential relevance in post-injury neurophysiology.

Metabolomic profiling in mouse models has further highlighted the impact of microbial metabolites on TBI (Du et al., 2021; Amaral et al., 2024). Interventions, i.e., probiotic administration and fecal microbiota transplantation (FMT), have shown potential to modulate the microbiota composition and improve neurological outcomes. For instance, in mouse models, FMT restored neurological function by influencing microbial metabolites and signaling pathways (Du et al., 2021; Wang et al., 2021). Similarly, probiotic treatment in another mouse study was associated with shifts in the microbiota community structure and increased markers of neuroprotection (Amaral et al., 2024). Collectively, these findings suggest that the gut microbiome may play a role in TBI recovery.

Consistent with the influence of microbial metabolites on TBI, studies have increasingly highlighted the changes of gut microbiota in the pathophysiology of TBI, with most research conducted using rodent models (Munley et al., 2023; Celorrio et al., 2024). In rat and mouse models, several studies have reported a reduction in alpha diversity of the gut microbiota following TBI, although some studies observed no significant differences (Munley et al., 2023; Celorrio et al., 2024). However, TBI consistently induces significant alterations in beta diversity across different studies, as shown by shifts in community composition, despite variability in the specific bacterial taxa affected. In a rat model of controlled cortical impact or fluid percussion injury, TBI causes acute dysbiosis of the gut microbiota characterized by reduced microbial diversity and notable decreases in butyrate-producing genera, e.g., *Agathobacter, Faecalibacterium*, and *Eubacterium* (Taraskina et al., 2022).

Research on the gut microbiota in human TBI is limited. An observational study in moderate-to-severe TBI patients admitted to a neurotrauma intensive care unit found high prevalence of *Proteobacteria* colonization as well as multidrug-resistant and colistin-resistant strains as early as 48 h post-injury, raising concerns about dysbiosis and infection risk (Mahajan et al., 2023). However, this study did not include taxonomic profiling of the gut microbiota. Evidence from other studies indicates that distinct and persistent alterations in the gut microbiota can persist for years after TBI (Urban et al., 2020; Pyles et al., 2024; Song et al., 2025).

Despite promising insights from animal models and limited human studies, a major barrier to translating gut microbiome research in TBI is the scarcity of human cohort studies, particularly in the early post-injury period. In this study, we characterized the gut microbiota profiles of 12 TBI patients and 13 healthy controls, none of whom had received antibiotics within 1 month prior to sample collection, by integrating high-resolution 16S rRNA sequencing with clinical metadata. Our analyses examined alterations in the diversity of the human gut microbiota associated with TBI, as well as potential functional changes in the gut microbiota of TBI patients within a few days after injury.

## Methods

### Recruitment of participants

Healthy volunteers without any known diseases and patients with TBI were recruited at Shanxi Provincial People's Hospital between February 2025 and June 2025, with one sample obtained per participant. For the TBI patients, 17 clinical variables were recorded, including antibiotic administration, Glasgow Coma Scale [GCS, an index to quantify severity (Bodien et al., 2021)], surgery, history of hypertension, diet, comorbidities, days since onset, and parameters indicative of inflammation, liver injury, coagulation activity, kidney function, and nutritional status. Except for surgical information, which was obtained during hospitalization, all other clinical data were collected on the same day as fecal sample collection.

### Inclusion and exclusion criteria

For TBI patients, eligibility required: (1) a confirmed diagnosis of TBI; (2) age between 18 and 80 years; (3) provision of fecal samples during hospitalization; (4) complete clinical records; and (5) written informed consent obtained from the patient or their legal representative. Healthy volunteers were eligible if they: (1) were aged 18–80 years; (2) reported no known diseases; and (3) provided written informed consent. Exclusion criteria included: (1) spontaneous intracerebral hemorrhage; (2) use of antibiotics within 30 days prior to fecal sample collection; (3) presence of gastrointestinal diseases; (4) pregnancy or lactation; and (5) death within 48 h of admission to the intensive care unit. The 30-day antibiotic-free window was selected as the best achievable practice in clinical settings.

### Sample collection and sequencing

Fresh stool samples were collected using a Microbiome Sample Collection Kit (Shanghai Personal Biotechnology Co., Ltd., China) and immediately placed in a −80 °C freezer. Samples were shipped on dry ice to Majorbio Co., Ltd. (China) for 16S rRNA sequencing. Briefly, DNA was extracted using the FastPure Stool DNA Isolation Kit (MJYH, Shanghai, China) and amplified with the primer pair 338F (5′-ACTCCTACGGGAGGCAGCAG-3′) and 806R (5′-GGACTACHVGGGTWTCTAAT-3′), targeting the V3-V4 region of the 16S rRNA gene. Sequencing libraries were prepared using the NEXTFLEX Rapid DNA-Seq Kit (Bioo Scientific, USA) and sequenced on the NextSeq 2000 platform with paired-end 300 bp reads.

### Pre-treatment of 16S rRNA sequencing data

Raw sequencing data were processed through a quality control pipeline involving sequence trimming with fasterq-dump, paired-end merging using FLASH, and removal of human-derived reads via Bowtie2. The resulting reads were aligned to the Greengenes2 database (https://greengenes2.ucsd.edu/) for taxonomic classification (McDonald et al., 2024). Species-level annotations were used where available. To minimize noise, taxa with relative abundances of at least 0.1% (or 0.01%) present in a minimum of 5% (or 15%) of samples were retained, as previously described (Wang et al., 2023), resulting in a total of 280 species-level taxa. All samples were sequenced to a depth exceeding 34,000 reads, and therefore, none were excluded due to insufficient quality.

### Diversity analysis

The 16S rRNA feature table was rarefied to a minimum sequencing depth of 34,000 reads per sample. Alpha diversity metrics, including Shannon index, observed taxa richness, and evenness, were calculated, with group comparisons conducted using two-sided Mann–Whitney *U*-tests. Beta diversity differences were visualized by Non-metric Multidimensional Scaling (NMDS) plots based on Bray-Curtis distance matrices and statistically tested using permutational multivariate analysis of variance (PERMANOVA) via the adonis2 function from the vegan R package (Dixon, 2003). The adonis2 function illustrates the effects of variables on the Bray-Curtis distance matrix or, in other words, the composition of the gut microbiota.

### Differential abundance analysis

Taxonomic differential abundance was evaluated using LefSe analysis implemented through the run_lefse function within the microbiomeMarker R package, which identified taxa that were statistically different between groups and estimated their effect sizes.

### Functional enrichment of KEGG orthologs

Based on the 16S rRNA profiles of the gut microbiota, PICRUSt2 (Douglas et al., 2020) was used to infer the abundance of KEGG orthologs (K numbers) by converting the 16S rRNA feature table into a KEGG ortholog feature table. Differential abundance analysis of K numbers between healthy and TBI patients was performed using DESeq2, a tool to analyze count-based data by modeling counts with negative binomial distributions, performing normalization, estimating dispersion, and identifying differentially expressed features between conditions (Love et al., 2014). Subsequently, K numbers were mapped to KEGG pathways according to the KEGG database annotations (Kanehisa et al., 2017). Enrichment of KEGG pathways was assessed through over-representation analysis utilizing the phyper function in R. The phyper function checked whether the number of genes found in a KEGG pathway was higher than what would be expected just by chance.

### Modeling of GCS scores using machine learning

Three missing entries in the diet variable were labeled as “unknown.” One missing value in the white blood cell variable was imputed by the mice function in R. The imputation procedure is widely used in machine learning modeling which fills in missing data by creating possible values based on the patterns in the rest of the dataset. All available data, including clinical and demographic features as well as 16S rRNA-based gut microbiota profiles, were standardized using the scale function in R. The scale function standardizes data by centering the values around the mean and scaling them by the standard deviation, so that each feature is on the same scale for fair comparison. The GCS score served as the response variable, with all other factors included as predictors. Models were built with the train function from the caret package (Kuhn, 2008) and evaluated via two-fold cross-validation repeated 20 times. Variable importance within each model, measuring how much each variable contributes to a model's predictions, was assessed using the varImp function in R. Model performance was summarized by the root mean square error, *R*^2^, mean absolute error, and their corresponding standard deviations. Nine algorithms were explored: tree-based eXtreme Gradient Boosting, k-nearest neighbors, random forest, boosted generalized linear model, conditional inference random forest, support vector machine with linear kernel, linear eXtreme Gradient Boosting, generalized linear model, and simple linear regression.

### Correlation analysis

Microbial abundances in the gut microbiota were normalized using the OTU table generated by the run_lefse function in R. Spearman's correlation, with *p*-values adjusted via the Benjamini–Hochberg method, was used to examine associations among demographic and clinical characteristics of TBI patients, as well as between these characteristics and abundances of the gut microbes.

## Results

### Profiles of the dataset

To investigate the role of the gut microbiota in TBI, fecal samples were collected from 13 healthy volunteers and 12 patients with TBI. No significant differences in age or sex were observed between the healthy and TBI groups (Table 1). The characteristics of these clinical variables are summarized in Table 2, and details are provided in Supplementary Datasets 1, 2. The average time of sample collection was 4.92 days after the initial visit (Table 2).

**Table 1:** Demographic characteristics of participants in the cohort.

**Characteristic**	***N***	**Overall *N* = 25^a^**	**Healthy *N* = 13^a^**	**TBI *N* = 12^a^**	***p*-value^b^**
Age	25	52 (16)	49 (14)	55 (18)	0.3
**Sex**	25				>0.9
Female		9 (36%)	5 (38%)	4 (33%)	
Male		16 (64%)	8 (62%)	8 (67%)	

^a^Mean (SD); n (%).

^b^Wilcoxon rank sum test; Fisher's exact test.

**Table 2:** Clinical characteristics of TBI patients in the cohort.

**Characteristic**	**Value^a^**
**Antibiotics**	
No	12 (100%)
**Glasgow coma scale**	
3	1 (8.3%)
6	1 (8.3%)
10	1 (8.3%)
11	1 (8.3%)
12	1 (8.3%)
14	3 (25%)
15	4 (33%)
**Surgery**	
Craniotomy hematoma evacuation	2 (17%)
Hematoma evacuation	1 (8.3%)
No	9 (75%)
**Hypertension history**	
No	8 (67%)
Yes	4 (33%)
**Diet**	
Liquid diet	2 (22%)
Regular diet	2 (22%)
Total parenteral formula	5 (56%)
Unknown	3 (33%)
**Comorbidity**	
Cerebral edema and hypoproteinemia	2 (17%)
Fracture	1 (8.3%)
Pulmonary infection and hypoproteinemia	2 (17%)
No	7 (58%)
Days since onset	4.92 (1.44)
White blood cell	7.383 (1.367)
Unknown	1 (11%)
Neutrophil percentage	77.85 (7.94)
Procalcitonin	0.22 (0.31)
C reactive protein	34.5 (36.8)
Aspartate transferase	24.71 (10.69)
Alanine transferase	22.7 (10.44)
D Dimer	5.53 (7.11)
Serum creatinine	56.5 (24.81)
Urea	5.12 (2.51)
Albumin	36.1 (3.82)

^a^Mean (SD); n (%).

Because antibiotics exert a strong influence on the composition of the human microbiota (Patangia et al., 2022), none of the participants in this study had received antibiotic treatment within 1 month prior to sample collection (Table 2). Therefore, potential effects attributable to antibiotic use were minimized.

Gut microbiota profiles from the 25 fecal samples were characterized using 16S rRNA gene sequencing. The minimum sequencing depth across all samples exceeded 34,000 reads (Supplementary Figure S1a). Alpha rarefaction analysis showed no significant difference in the number of observed bacterial taxa when rarefied to 34,000 vs. 32,000 reads (Supplementary Figure S1b). Consequently, all samples were rarefied to 34,000 reads for subsequent diversity analyses.

### Association between diversity of the gut microbiota and TBI

**Figure 1:**
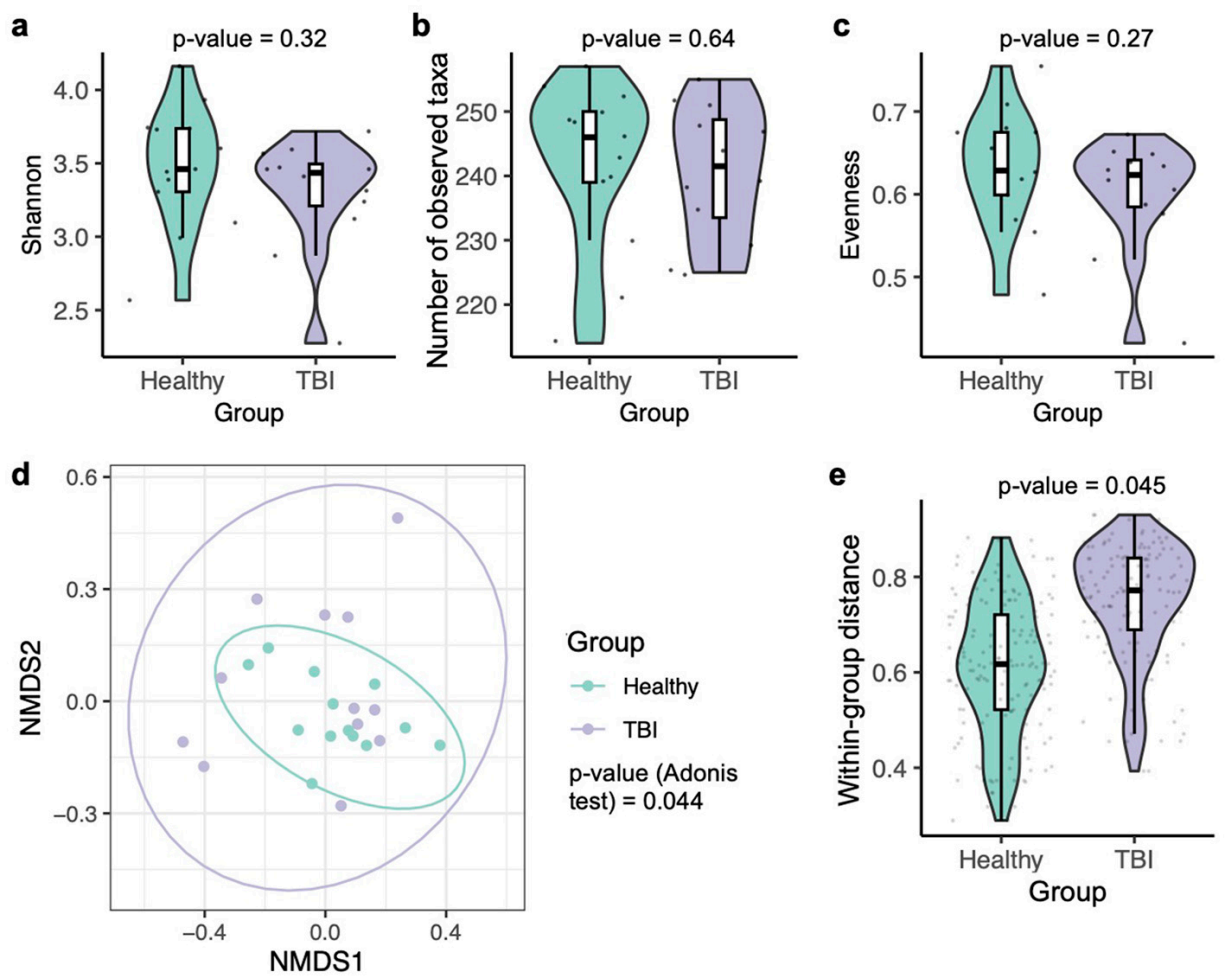
Gut microbiota diversity and compositional differences associated with TBI. Alpha diversity metrics, including Shannon index **(a)**, observed taxa **(b)**, and evenness **(c)**, are shown for healthy and TBI groups, with statistical comparisons performed using two-sided Mann–Whitney *U*-tests. **(d)** Differences in overall microbiota composition are illustrated using a NMDS plot based on Bray-Curtis distances and evaluated with the Adonis test. **(e)** Within-group Bray-Curtis distances are compared between healthy and TBI individuals to assess within-group variability, using the two-sided Mann–Whitney *U*-test.

In rodent models, multiple investigations have shown that TBI can lead to decreased alpha diversity in the gut microbiota, whereas other studies have found no notable changes (Munley et al., 2023; Celorrio et al., 2024). In the present human cohort, no significant differences in alpha diversity, i.e., Shannon index, observed taxa richness, and evenness, were observed between healthy controls and TBI patients (Figures 1a–c). In contrast, consistent with findings from animal studies (Munley et al., 2023; Celorrio et al., 2024), the Adonis test revealed a significant association between beta diversity of the gut microbiota and TBI status (Figure 1d). Specifically, samples from the healthy group exhibited tighter clustering on the NMDS plot compared to those from the TBI group (Figure 1d), and the within-group similarity quantified by the Bray-Curtis distance was significantly lower among healthy controls (Figure 1e). These results imply that TBI could disrupt the homogeneity of the gut microbiota, resulting in increased inter-individual variability in the composition of the gut microbiota.

This increased dispersion in beta diversity among the TBI patients may be influenced by variability in treatment regimens. To investigate this, an Adonis test incorporating multiple clinical variables using a marginal model was conducted on the 12 TBI samples. The effect of each variable on the gut microbiota was tested marginally, i.e., controlling for all the other variables included in the model. As indicated by the significance “Pr(>*F*),” none of the tested factors, i.e., sex, age, surgery, hypertension history, diet, or comorbidity, had a significant effect on beta diversity (Supplementary Table S1). Thus, the tested variables may have had only a limited influence on the composition of the gut microbiota in this study, likely due to the small number of cases.

### Differential abundance of gut microbes in TBI patients

**Figure 2:**
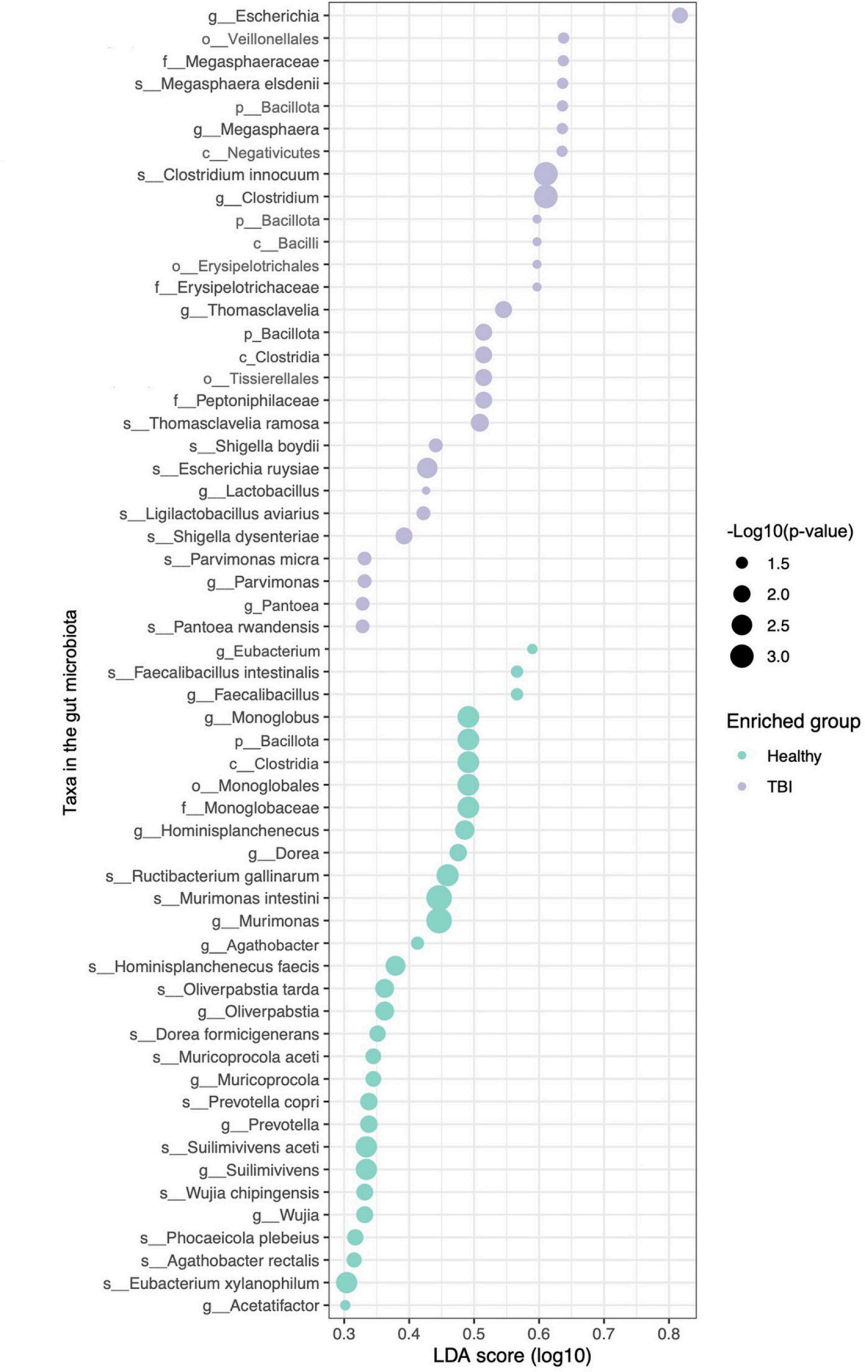
Differential gut microbiota composition associated with TBI. LefSe analysis was used to identify bacterial taxa differentially enriched between healthy controls and TBI patients. The LDA score reflects the effect size of each taxon, indicating its contribution to group differences. Dot size corresponds to statistical significance as computed by LefSe, and dot color indicates the group in which each taxon is enriched.

Differential abundance analysis was performed using the Lefse method (Segata et al., 2011), revealing distinct differences in the abundance of bacterial taxa between TBI patients and healthy controls (Figure 2). As introduced above, SCFA is a key metabolite that maintains neurological and psychiatric health (Guo et al., 2022; Lan et al., 2024) and SCFA supplementation has a potential protective role mediated via the gut-brain axis in a TBI mouse model (Taraskina et al., 2022; Celorrio et al., 2025). Consistently, taxa enriched in healthy individuals included members of the *Lachnospiraceae* family, e.g., *Eubacterium, Agathobacter*, and *Faecalibacillus intestinalis*, which are recognized for their production of SCFAs like butyrate and propionate (Guo et al., 2022; Lan et al., 2024). Thus, the reduced abundance of SCFA-producing taxa in TBI patients may impair gut-brain axis homeostasis, thereby potentially aggravating neuroinflammation and neurological dysfunction associated with TBI.

In contrast, *Escherichia* and *Shigella* species were among the most enriched taxa in the TBI group (Figure 2). These genera are generally considered opportunistic and are frequently associated with gut dysbiosis and intestinal inflammation (Baldelli et al., 2021; Li et al., 2022). Such microbial imbalances can exacerbate systemic inflammation and contribute to adverse neurological outcomes (Munley et al., 2023). Collectively, these findings indicate that TBI induces dysbiosis within the human gut microbiota.

Additionally, among taxa enriched in the TBI group, species in the genus of *Lactobacillus* are well-known producers of GABA, a key inhibitory neurotransmitter involved in neuroimmune regulation and gut-brain axis signaling (Figure 2) (Ma et al., 2019; Bin-Khattaf et al., 2022; Conn et al., 2024). Because neuroprotective effects of *Lactobacillus* have been demonstrated in mouse models of TBI through the modulation of inflammation, the increased abundance of *Lactobacillus* in TBI patients may reflect a compensatory microbiome response to neural injury and stress, potentially aimed at modulating gut-brain axis communication. However, contrasting findings show that TBI can also lead to a transient reduction in *Lactobacillus*, particularly *L. gasseri*, during the early post-injury phase in murine models (Treangen et al., 2018).

Together, these findings suggest that TBI resulted in gut dysbiosis characterized by the depletion of SCFA production-associated microbes and the overgrowth of dysbiosis-associated taxa, e.g., *Escherichia*. However, elevated *Lactobacillus* in TBI patients may serve as a compensatory mechanism. Whether shifts in the gut microbiota, particularly in taxa involved in SCFA and GABA pathways, impact the gut-brain axis during head injury and recovery remains to be elucidated.

### Functional enrichment of KEGG orthologs and pathways in the gut microbiota associated with TBI

To further explore the metabolic potential of the gut microbiota, functional profiles were inferred from 16S rRNA sequencing data by predicting the abundance of KEGG Orthologs (K numbers) using the PICRUSt2 software (Douglas et al., 2020). Differential enrichment of specific K numbers was assessed using the DESeq2 software (Love et al., 2014). Subsequently, each enriched K number was mapped to corresponding KEGG pathways, and pathway-level enrichment was evaluated through over-representation analysis (Wieder et al., 2021).

**Figure 3:**
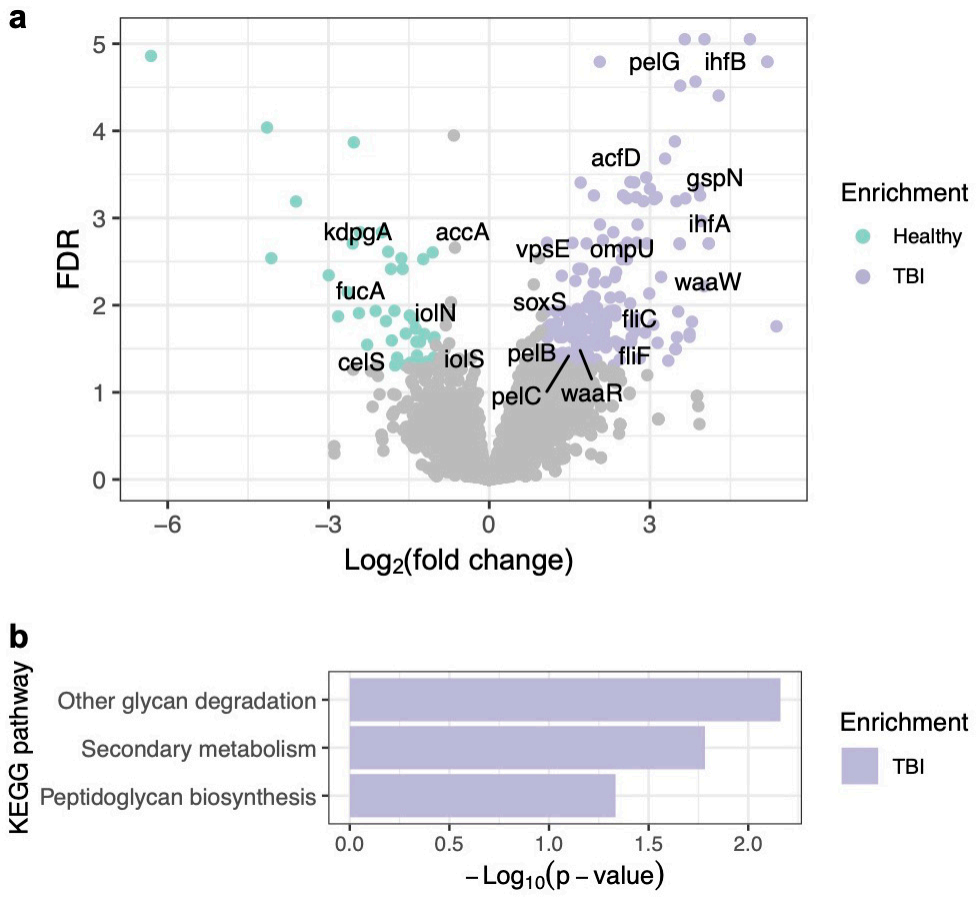
Functional enrichment of KEGG orthologs (K numbers) and pathways in the gut microbiota associated with TBI. **(a)** K numbers, representing KEGG orthologs (functionally similar genes across species), were quantified using PICRUSt2. Differential abundance analysis of K numbers between healthy and TBI groups was performed using DESeq2. The volcano plot illustrates both the magnitude of change (log_2_ fold change) and statistical significance (FDR) for each K number. Selected significantly altered K numbers are labeled with their corresponding gene names. More details are shown in Supplementary Dataset 3. **(b)** Over-representation analysis was conducted to identify KEGG pathways significantly enriched in the gut microbiota of either healthy controls or TBI patients based on differentially abundant K numbers. More details are shown in Supplementary Dataset 4.

There were 43 and 211 K numbers significantly enriched in the gut microbiota of healthy controls and TBI patients, respectively (Figure 3a and Supplementary Dataset 3). Consistent with the differential abundance analysis described above, several K numbers (annotated as matched gene names in Figure 3a) encode key enzymes catalyzing rate-limiting or critical steps in metabolic pathways leading to SCFA production, including *fucA* (Cheong et al., 2022), *accA* (Cheng et al., 2023), *celS* (Liao et al., 2024), *iolN/iolS* (Yoshida et al., 2008), and *kdpgA* (Reher et al., 2010), all enriched in the healthy group. For example, *fucA*, involved in fucose metabolism, has been shown to promote SCFA and 1,2-propanediol production (Cheong et al., 2022). The *accA* gene encodes the α-subunit of acetyl-CoA carboxylase, which catalyzes the conversion of acetyl-CoA to malonyl-CoA, the first committed step in fatty acid synthesis (Soriano et al., 2006). In microbial community studies, increased abundance of *accA* has been positively correlated with enhanced SCFA production (Cheng et al., 2023). Furthermore, several key genes directly responsible for SCFA production, such as *pccA* and *mcmA*, showed reduced but nonsignificant abundance changes in the TBI group, while others, e.g., *ackA* and *pta* (Frolova et al., 2022), showed no significant difference between groups. Overall, the enrichment of multiple upstream and regulatory SCFA-associated genes in healthy participants suggests a greater potential for SCFA biosynthesis capacity in this group compared to TBI patients.

In contrast, many K numbers with matched genes associated with biofilm formation, a key virulence factor in the gut microbiota (Palandurkar and Kumar, 2023; Singh et al., 2025), were enriched in the TBI group (Figure 3a). These included genes for exopolysaccharide biosynthesis, such as *pelB, pelC*, and *pelG* (Amyx-Sherer and Reichhardt, 2024), as well as *ompU, acfD, vpsE*, and *gspN* (Teschler et al., 2022). Genes involved in flagellar motility, e.g., *fliF* and *fliC*, which facilitate initial surface attachment (Guttenplan and Kearns, 2013), and LPS core biosynthesis genes *waaR* and *waaW*, which influence biofilm stability (Aquilini et al., 2010), were also more abundant in the TBI group. Furthermore, global regulators *ihfA* and *ihfB* (Fan et al., 2023) and transcriptional regulators *rob, marA, soxS*, and *ramA* (Holden and Webber, 2020; Middlemiss et al., 2023), which modulate biofilm-related genes and stress responses, were enriched. Such enrichment implies a microbial community in TBI patients with an enhanced capacity for adhesion, matrix production, and persistence within host environments, potentially contributing to chronic inflammation or impaired recovery.

At the pathway level, none of the KEGG pathways remained significant after multiple-testing correction (Supplementary Dataset 4). However, nominal enrichment (*p* ≤ 0.05) revealed that three pathways, i.e., secondary metabolism, peptidoglycan biosynthesis, and other glycan degradation, were enriched in the TBI group (Figure 3b), consistent with a specific set of differentially abundant genes (FDR ≤ 0.05, Supplementary Dataset 3) identified at the KEGG Ortholog level. Genes involved in secondary metabolism, including *thnK, thnN*, and *pltB*, are responsible for the biosynthesis of naturally produced antimicrobial compounds such as thienamycin (Rodríguez et al., 2008) and pyoluteorin (Nowak-Thompson et al., 1999). Their enrichment implies an increased production of bioactive molecules that may enable microbes to outcompete others or adapt to altered host immune responses. The enrichment of the peptidoglycan biosynthesis pathway, marked by genes like *murE, bacA, mraY*, and *pbpA*, indicates elevated synthesis of peptidoglycan, a major component of bacterial cell walls especially in both Gram-positive and Gram-negative bacteria (Garde et al., 2021). Additionally, the upregulation of glycan degradation genes, including *hex, manB*, and *afcA*, indicates an adaptive microbial response to exploit host-derived glycans as alternative nutrient sources, potentially triggered by changes in nutrient availability in TBI patients. A shared feature in secondary metabolism and glycan degradation pathways is their upregulation in response to nutrient limitation or stress environment. This pattern may correspond with reduced food intake and altered nutrient availability in the gastrointestinal tract of TBI patients.

### Predicting GCS scores from gut microbiota profiles and clinical-demographic factors

**Figure 4:**
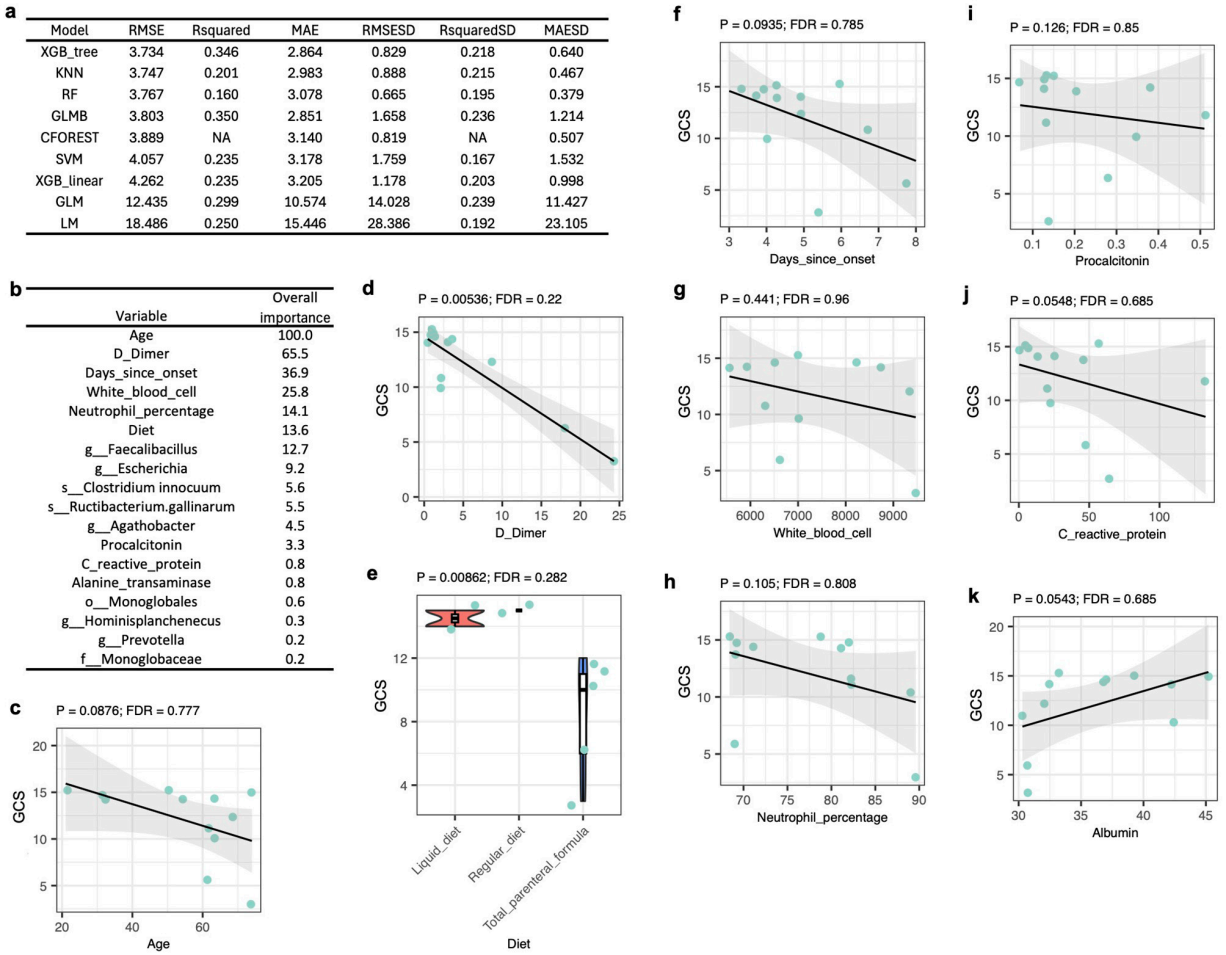
Predicting GCS scores from tut microbiota profiles and clinical-demographic factors. **(a)** Nine machine learning algorithms were employed to predict GCS scores, a clinical measure of consciousness level following brain injury. Model performance was assessed through two-fold cross-validation repeated 20 times. Performance metrics included root mean square error (RMSE), *R*-squared (*R*^2^), mean absolute error (MAE), and their respective standard deviations (RMSESD, RsquaredSD, MAESD). Algorithms used include: tree-based eXtreme Gradient Boosting (XGB_tree), k-nearest neighbors (KNN), random forest (RF), boosted generalized linear model (GLMB), conditional inference random forest (CFOREST), support vector machine with linear kernel (SVM), linear eXtreme Gradient Boosting (XGB_linear), generalized linear model (GLM), and simple linear model (LM). **(b)** The variable importance ranking from the XGB_tree model illustrates the relative influence of each feature on predictive performance. **(c–k)** Correlations among clinical or demographic variables were quantified by Spearman's rank correlation (see details in Supplementary Dataset 6). *p*-Values were adjusted for multiple testing using the Benjamini–Hochberg method to control the false discovery rate (FDR). Associations between GCS scores and each clinical or demographic variable included in the XGB_linear model are shown.

A GCS score is essential to identify the severity of TBI. To further understand the role of gut microbes in TBI, all the data involved in this study for the 12 TBI patients were applied to establish predictive modeling of GCS scores using a 10-times repeated two-fold cross-validation strategy. Nine different algorithms were applied and, based on the root mean square error values, the tree-based eXtreme Gradient Boosting (XGB tree) algorithm had the best performance (Figure 4a). More specifically, this XGB tree model achieved a root mean square error of 3.734, indicating that, on average, its predictions deviated from the observed values by about 3.7 units. The mean absolute error was 2.864, suggesting that the average magnitude of prediction errors, without considering direction, was approximately 2.86 units. The *R*^2^ value of 0.346 indicated that the model explained roughly 34.6% of the variance in the observed GCS scores, implying moderate predictive performance with substantial unexplained variability. Therefore, it appears that the primary factors accounting for the remaining 65% of the variance in GCS scores were not captured in the dataset, potentially including the degree of injury at the time of hospitalization and biomarkers that directly reflect the status of the nervous, cardiovascular, respiratory, immune, and other related systems.

It is worth noting that most decision tree-based algorithms, e.g., XGB tree and random forest, demonstrated better performance than linear models, like linear eXtreme Gradient Boosting, generalized linear model, and simple linear regression (Figure 4a). Hence, the relationships between the predictors and GCS scores were likely nonlinear and tree-based methods were inherently better suited to capture than linear approaches.

The contribution of each factor in the XGB tree model was evaluated using feature importance, which quantifies how much each predictor improves model performance, measured by gain, cover, and frequency, across all trees in the ensemble (Figure 4b). Consistent with the differential abundance analysis (Figure 2), all the bacterial taxa contributing to the XGB tree model are the gut microbes that had significant differential abundances between healthy controls and TBI patients. Particularly, *Clostridium innocuum* and the genus of *Escherichia* had increased abundance in TBI patients, but all other taxa were enriched in healthy participants (Figures 2, 4b). Consistent with these observations, a rat model demonstrated that head injury led to a dramatic increase in *Escherichia coli* populations (Waligora-Dupriet et al., 2018), and *C. innocuum* has been associated with gut inflammation and disease (Cherny et al., 2021).

Spearman's correlation analysis was used to evaluate the relationships between clinical-demographic factors and GCS scores (Supplementary Dataset 5). Consistent with previous studies showing that older patients tend to experience more severe TBI (Hukkelhoven et al., 2003), age had the strongest influence on GCS scores among the tested factors and was negatively, though not significantly, correlated with GCS (Figure 4c). Other clinical variables included in the XGB tree model, i.e., D-dimer, total parenteral nutrition, days since injury onset, white blood cell count, neutrophil percentage, procalcitonin, and C-reactive protein, were also associated with greater TBI severity (Figures 4d–j). Conversely, albumin levels were higher in patients with better GCS scores (Figure 4k), although many of these correlations did not reach statistical significance. These clinical markers reflect inflammatory and immune responses, as well as metabolic and nutritional status. Elevated D-dimer levels have been linked to increased coagulation and potential microvascular injury (Weitz et al., 2017), while higher procalcitonin and C-reactive protein, together with lower albumin levels, indicate systemic inflammation and possible infection (Karaca et al., 2023). Alterations in white blood cell counts and neutrophil percentages further imply immune activation (Abramson and Melton, 2000; Buonacera et al., 2022). Moreover, the need for total parenteral nutrition and longer time since injury may reflect greater physiological stress and impaired recovery. Collectively, these findings highlight how TBI induces widespread systemic effects, including alterations in the gut microbiota, that extend beyond the central nervous system.

Additionally, Spearman's correlations between clinical-demographic characteristics of the TBI patients and the abundances of the gut microbial taxa were evaluated (Supplementary Dataset 6). The only significant outcome after multiple correction of the *p*-values was the positive correlation between the species of *Wujia chipingensis* and albumin (Supplementary Figure S2). Aligned with this observation, *W. chipingensis* was enriched in healthy participants (Figure 2) and albumin was associated with lower levels of severity of TBI (Figure 4k). Additionally, *W. chipingensis* has been associated with lower levels of C-reactive protein levels, indicating its potential involvement in modulating inflammatory responses (Zhu et al., 2025). Consistent with the neuroprotective effects of *Lactobacillus* in mouse models (Ma et al., 2019; Bin-Khattaf et al., 2022; Conn et al., 2024), *Lactobacillus* was significantly negatively correlated with C-reactive protein, a well-established inflammatory biomarker, prior to *p*-value adjustment (Supplementary Dataset 6).

## Discussion

In this study, our findings showed that TBI in humans was associated with alterations in gut microbial composition and potential biological function, characterized by depletion of SCFA-producing taxa and enrichment of taxa commonly associated with dysbiosis. SCFAs are well-characterized mediators of gut barrier integrity, immune regulation, and gut-brain signaling (Guo et al., 2022; Lan et al., 2024; Celorrio et al., 2025). The reduced SCFA biosynthetic potential in TBI patients could therefore contribute to increased gut permeability, systemic inflammation, and exacerbation of neuroinflammatory cascades after TBI. Functionally, the enrichment of KEGG orthologs linked to biofilm formation, secondary metabolism, and glycan degradation in the TBI group further implies that post-injury microbial communities may be more adhesive, stress-tolerant, and capable of exploiting host-derived glycans under nutrient-limited conditions, features that can promote persistence and inflammation in injured hosts.

GABA, an inhibitory neurotransmitter involved in neuroimmune regulation and gut-brain communication, plays a protective role in TBI (Mazzoli and Pessione, 2016; Duranti et al., 2020). The observed enrichment of *Lactobacillus* in TBI patients suggests a potential increase in gut GABA, which could represent a compensatory response to injury-induced dysbiosis or a transient bloom influenced by diet, hospitalization, or other treatments (Ma et al., 2019; Bin-Khattaf et al., 2022; Conn et al., 2024). Supporting this idea, previous research has reported an elevated GABA-to-glutamate ratio in the gut microbiota as a metabolic signature in mild autism spectrum disorder, linked to overrepresentation of *Escherichia* (Wang et al., 2025) and our study found *Escherichia* to be enriched in the TBI group. Furthermore, *Lactobacillus* is enriched in schizophrenia, accompanied by changes in the glutamate-glutamine-GABA cycle (Nuncio-Mora et al., 2023). Together, these findings highlight the need for further investigation into GABA production by the gut microbes and their potential role in TBI recovery.

Linking microbial signatures and clinical variables to the severity of TBI, our predictive modeling (XGB tree) explained a modest fraction of GCS variance (*R*^2^ ≈ 0.35), indicating that gut microbiota and the clinical-demographic factors measured here capture part but not the majority of determinants of consciousness after injury. Several clinical markers associated with inflammation, coagulation, and nutritional status, e.g., D-dimer, procalcitonin, CRP, albumin, correlated with GCS, consistent with a model in which systemic inflammation and metabolic derangement after TBI influence gut microbial composition. Notably, albumin was positively associated with *W. chipingensis*, a taxon enriched in healthy participants, and with better GCS scores, implying that preserved nutritional status may mitigate dysbiosis of the gut microbiota.

This study has several important limitations. The sample size is small and includes only a single time point, which limits the statistical power of our analyses. Consequently, the detection of correlations between bacterial taxa and clinical variables and detecting pathway-level changes were constrained, particularly after correcting for multiple comparisons. Detection of alpha diversity associated with TBI could potentially be limited by the small sample size. Additionally, despite efforts to exclude recent antibiotic exposure, the cohort remains heterogeneous in terms of injury timing, clinical management (including nutrition and surgery), disease severity, and potentially probiotic and dietary factors, which may limit the generalizability of these findings to broader populations.

Taxonomic resolution from 16S rRNA sequencing is limited at the strain level and precludes definitive assignments of pathogenic vs. beneficial roles for taxa within polyphyletic genera, e.g., *Clostridium*. Achieving strain-level resolution requires metagenomic sequencing, and functional inferences based on predicted KEGG orthologs likewise need validation through metabolomic measurements or other quantification methods (e.g., direct quantification of SCFAs, GABA, and biofilm-related products).

It was challenging to collect fecal samples from patients with severe TBI who had not received antibiotics, due to several factors, e.g., constipation, critical condition, fasting, reduced stool output associated with total parenteral nutrition, and prophylactic antibiotic use in patients with open or penetrating TBI. Consequently, seven of the 12 TBI patients included in this study presented with only mild impairment of consciousness (GCS score ≥ 13, Table 2), which may introduce bias in sample collection with respect to disease severity.

In conclusion, our results indicate that human TBI is associated with a shift in gut microbial composition away from SCFA-producing, homeostatic taxa toward communities enriched in opportunistic, adhesive, and potentially pro-inflammatory organisms. Elucidating causal relationships and therapeutic opportunities will require larger, higher-resolution, and longitudinal investigations.

## Data Availability

Raw 16S rRNA sequencing data are available in the Genome Sequence Archive database belonging to China National Center for Bioinformation (accession code: PRJCA045145).
