# Supplementary material for: Gut microbiota and traumatic brain injury: insights from an antibiotic-free cohort
Source: Front Microbiol. 2026 Jan 2;16:1697206. doi: 10.3389/fmicb.2025.1697206 (PMC12808345; doi:10.3389/fmicb.2025.1697206)
Supplement: Supplementary file 2 [file Presentation_1.pdf]

Table S1. Results of adonis analysis incorporating multiple clinical variables in 12 TBI Samples. Age was dichotomized based on the median value, and missing data were categorized as "unknown." Statistical outcomes are presented. Df is the degrees of freedom for each factor, SumOfSqs is the variance in the distance matrix attributed to that factor, R^2 is the proportion of variance explained, F is the pseudo-F statistic comparing between-group to within-group variation, and Pr(>F) is the permutation-based p-value indicating statistical significance.

	Df	Sum Of Sqs	R2	F	Pr(>F)
Sex	1	0.34	0.11	1.02	0.45
Age	1	0.26	0.08	0.78	0.72
Surgery	2	0.45	0.14	0.68	0.83
hypertension_history	1	0.49	0.15	1.46	0.23
Diet	2	0.73	0.23	1.10	0.45
Comorbidity	3	0.87	0.27	0.87	0.66
Residual	1	0.33	0.10	NA	NA
Total	11	3.17	1.00	NA	NA

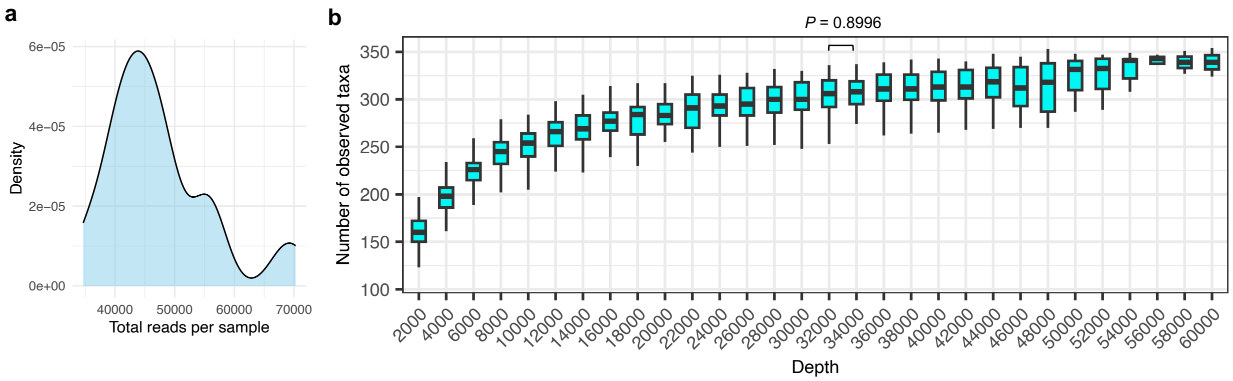

Fig. S1 **Sequencing depth of microbiota profiles. (a)** After quality control, trimming, merging of paired sequence reads, and removal of human reads, the total reads for the 25 gut microbiota samples are displayed. **(b)** Alpha rarefaction of the 25 samples indicates that a total sample reads threshold of 34,000 is suitable for subsequent analyses. Differences between conditions were assessed using the two-sided Mann–Whitney U test.

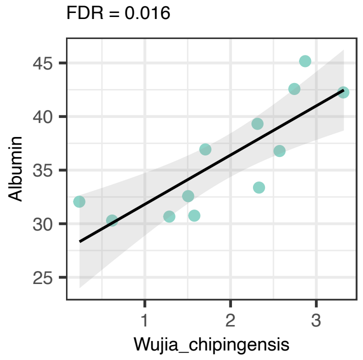

Fig. S2 Correlation between albumin and the abundance of Wujia chipingensis in the gut microbiota. Correlations between demographic and clinical characteristics of TBI participants and the abundances of the gut microbial taxa were quantified by the Spearman's rank correlation with p-values adjusted using the Benjamini-Hochberg method to control the false discovery rate

(FDR). Details are listed in Supplementary Dataset 4. The correlation between albumin and the abundance of Wujia chipingensis in the gut microbiota is illustrated.
